# miR-7b-3p Exerts a Dual Role After Spinal Cord Injury, by Supporting Plasticity and Neuroprotection at Cortical Level

**DOI:** 10.3389/fmolb.2021.618869

**Published:** 2021-03-31

**Authors:** Matilde Ghibaudi, Marina Boido, Darrell Green, Elena Signorino, Gaia Elena Berto, Soraya Pourshayesteh, Archana Singh, Ferdinando Di Cunto, Tamas Dalmay, Alessandro Vercelli

**Affiliations:** ^1^Department of Neuroscience “Rita Levi Montalcini,” Neuroscience Institute Cavalieri Ottolenghi, University of Turin, Orbassano, Italy; ^2^Polymers and Biomaterials, Italian Institute of Technology, Genova, Italy; ^3^Norwich Medical School, University of East Anglia, Norwich, United Kingdom; ^4^School of Biological Sciences, University of East Anglia, Norwich, United Kingdom

**Keywords:** miRNAs, spinal cord injury, axon regeneration, sprouting, neuronal development, Wipf2

## Abstract

Spinal cord injury (SCI) affects 6 million people worldwide with no available treatment. Despite research advances, the inherent poor regeneration potential of the central nervous system remains a major hurdle. Small RNAs (sRNAs) 19–33 nucleotides in length are a set of non-coding RNA molecules that regulate gene expression and have emerged as key players in regulating cellular events occurring after SCI. Here we profiled a class of sRNA known as microRNAs (miRNAs) following SCI in the cortex where the cell bodies of corticospinal motor neurons are located. We identified miR-7b-3p as a candidate target given its significant upregulation after SCI *in vivo* and we screened by miRWalk PTM the genes predicted to be targets of miR-7b-3p (among which we identified *Wipf2*, a gene regulating neurite extension). Moreover, 16 genes, involved in neural regeneration and potential miR-7b-3p targets, were found to be downregulated in the cortex following SCI. We also analysed miR-7b-3p function during cortical neuron development *in vitro*: we observed that the overexpression of miR-7b-3p was important (1) to maintain neurons in a more immature and, likely, plastic neuronal developmental phase and (2) to contrast the apoptotic pathway; however, in normal conditions it did not affect the Wipf2 expression. On the contrary, the overexpression of miR-7b-3p upon *in vitro* oxidative stress condition (mimicking the SCI environment) significantly reduced the expression level of Wipf2, as observed *in vivo*, confirming it as a direct miR-7b-3p target. Overall, these data suggest a dual role of miR-7b-3p: (i) the induction of a more plastic neuronal condition/phase, possibly at the expense of the axon growth, (ii) the neuroprotective role exerted through the inhibition of the apoptotic cascade. Increasing the miR-7b-3p levels in case of SCI could reactivate in adult neurons silenced developmental programmes, supporting at the same time the survival of the axotomised neurons.

## Introduction

Around 6 million people worldwide live with a significant disability caused by traumatic spinal cord injury (SCI), which is associated with devastating social impact plus huge economic cost (Singh et al., 2014). Immediately after a SCI event, timely surgical intervention is critical to stabilise the lesion. The glial scar that forms at the site of the lesion produced by reactive astrocytes delimiting the area becomes a temporal obstacle for axon regeneration. When therapeutic strategies are applied at later stages, they are generally ineffective. The acute/intermediate phase when the formation of glial scar is not yet complete is an appropriate window to efficiently promote the regeneration process (Tran et al., 2018). Motor disabilities that follow SCI trauma are essentially due to axotomised corticospinal fibres [corticospinal motor neurons (CSMNs) whose cell body is located in the layer V of the cortex] that are unable to re-establish functional connections. Despite CSMN attempts to regenerate, their efforts fail because of a non-permissive regrowth environment, neurotrophic factor deprivation and inhibitory myelin-associated molecules (Brown and Martinez, 2019; Xu A.K. et al., 2019). No successful treatment is available for SCI patients although several therapeutic interventions such as cell engraftment, 3D scaffolds and gene therapy are currently under investigation. These interventions aim to reduce glial scar formation and to promote neuronal regeneration (Assinck et al., 2017; Boido et al., 2019; Cofano et al., 2019; Zhang et al., 2019).

In the last two decades, a class of small RNA (sRNA) gene regulatory molecules termed microRNAs (miRNAs) emerged as promising targets in SCI. miRNAs are transcripts ∼22 nucleotides in length that bind to the 3′UTR of target messenger RNA (mRNA) leading to translational inhibition (Green et al., 2016; Pinchi et al., 2019). miRNAs are abundantly expressed in the central nervous system (CNS) with specific temporal and spatial patterns contributing to highly accurate control of gene expression both in physiological and pathological conditions (Cao et al., 2016). As a consequence of SCI, miRNAs purportedly undergo a sustained change in their expression profile leading to downstream gene regulatory effects. Potential of miRNAs in the context of SCI lies in the double possibility to exploit them as diagnostic markers and to manipulate their expression through a mimic/antagomir strategy (Almurshidi et al., 2019). As key mediators of several neuronal processes both in the brain and spinal cord, miRNAs are commonly classified into specific functional groups depending on the role they are exerting. Entire miRNA families or clusters have been attributed to selectively regulate axon guidance and outgrowth pathways (e.g., miR-430 family and miR-17-92 cluster) at developmental levels as well as to modulate the inflammation, proliferation, neuroprotection, apoptosis, and regeneration processes after SCI (Nieto-Diaz et al., 2014; Ning et al., 2014). Immediately after injury, there is a significant increase in the expression of regeneration and neuroprotection associated genes, a phenomenon controlled by the early activation of specific transcriptional factors that are regulated by several miRNAs. For example, miR-21, miR-29, and miR-199 (acting on the PTEN/mTOR pathway) have been already described to influence axonal regrowth after SCI (Ning et al., 2014; Sun et al., 2018). Strategies that specifically target one single miRNA or a specific set of miRNAs may promote functional recovery after SCI (Ghibaudi et al., 2017; Shi et al., 2017). Although the list of miRNAs functionally involved in axonal regrowth/plasticity is extensive in the literature, there is a lack of experimental evidence investigating miRNA networks acting on these processes *in vivo*. The majority of non-coding RNAs investigated in SCI studies are generally focused only on the spinal cord, disregarding the cerebral cortex where CSMNs reside. These cells undergo a structural remodelling within the cortex that can have a significant impact on the molecular mechanisms driving the processes after a SCI lesion manifests.

Global identification of sRNAs via library construction and next generation sequencing is biassed for sequences that can readily anneal to adapters with a fixed sequence. miRNAs that have a lower annealing efficiency are less likely to be ligated to adapters and less probable to be sequenced. To enhance characterisation of the miRNA population, we used high definition (HD) adapters that contain four degenerate assigned nucleotides at the ligating ends of HiSeq 2500 adapters. We profiled miRNAs at the cortical level in the acute phase following a traumatic SCI in order to characterise those involved in the regeneration and neuroprotection processes. We investigated the sensorimotor cortex of both young and adult mice when neuronal networks are still refining [Postnatal 15 (P-15)] and when the CNS is considered mature and less plastic (postnatal day 90 or P-90) so to establish an age-related effect. We defined two time points 12 h and 3 days after SCI in order to identify those miRNAs acting in the primary and secondary phase when the therapeutic approaches are more effective. We observed an upregulation of miR-7b-3p in the cortex of SCI mice. To better understand the role of this miRNA, we carried out a number of *ex vivo* and *in vitro* experiments, finally suggesting that the regulation of miR-7b-3p could be exploited as a therapeutic target to promote axon plasticity.

## Materials and Methods

### SCI Mouse Model

C57BL/6J male mice were purchased from Envigo (Udine, Italy). Animals were maintained under standard conditions with free access to food and water. All experimental procedures on live animals were performed according to the European Activities Communities Council Directive of 86/609/EEC 1986 and University of Turin institutional guidelines on animal welfare (authorisation number 17/2010-B). P-15 and P-90 mice were divided into two groups: SCI mice (*n* = 55) and sham controls (*n* = 61). Mice were anaesthetised and injured as previously described (Boido et al., 2009). Briefly, the cervical spine was exposed and spinal muscles were displaced laterally. The lesion was performed by exposing the entire spinal cord and using a 27-gauge needle, transected at C6 level. In the SHAM group, the spinal cord was exposed without any damage.

### Histological Analysis

A set of animals (P15 *n* = 15, P90 *n* = 14) was employed for the histological analysis of the sensorimotor cortex in order to better characterise the SCI model. Either 12 h or 3 days after injury both P-15 and P-90 mice were anaesthetised with 3% isoflurane vaporised in O_2_/N_2_O 50:50 and transcardially perfused with 0.1 M PBS, pH 7.4 followed by 4% PFA in PBS. The brain and spinal cord (C6 level) were dissected and post-fixed for 2 h at 4°C in the same fixative solution. Samples were transferred overnight into 30% 0.1M PBS at 4°C then embedded in cryostat medium (Killik, Bio-Optica, Milan, Italy), frozen at −70°C in 2-methylbutane and cut on the cryostat (Microm HM 550) in coronal and transverse 50 μm thick sections for brain and spinal cord, respectively. Sections were collected into 1× PBS prior to immunofluorescence reactions and Fluoro-Jade C (FJC; Histo-Chem Inc., Jefferson, AR, United States) staining.

### Immunofluorescence

For evaluating astrogliosis and microglia activation, sections were immunolabelled with GFAP and IBA1, respectively. Briefly, after 30 min in PBS-triton 2% and 1 h in blocking solution (0.2% Triton X-100 and 10% normal donkey serum in PBS pH 7.4) (NDS; Sigma-Aldrich, Milan, Italy), sections were incubated with primary antibodies (IBA1, Wako Laboratories Chemicals, 019-09741, Japan, 1:500; GFAP, Dako Cytomation, Z0334, Denmark, 1:500) in the same solution at 4°C overnight. Then the sections were washed in 1× PBS and incubated with the secondary antibody (Jackson Immuno Research Laboratories; 1:200 donkey anti-rabbit cyanine 3-coniugated). Images of the sensorimotor cortex were taken with Nikon DS-5Mc digital camera on a Nikon Eclipse 80i epifluorescence microscope. Photomicrographs at 40× magnification were corrected for contrast and brightness enhancement with ImageJ.

### Fluoro-Jade C Staining

To stain degenerating neurons, sections were treated for FJC staining (Histo-Chem Inc., Jefferson, AR, United States), following supplier’s instructions. Brain and spinal cord sections were mounted on 2% gelatin coated slides and dried overnight at RT. The following day, sections were immersed in a solution of 1% sodium hydroxide in 80% ethanol for 5 min. Then they were rinsed for 2min in 70% ethanol, 2 min in distilled water and then incubated in 0.06% potassium permanganate solution in 1× PBS for 10 min. Then slides were rinsed for 2 min in distilled water and transferred for 20 min to a 0.0004% solution of FJC dissolved in 0.1% acetic acid. The sections were rinsed in distilled water three times for 1 min before drying at 37°C for ∼30 min. Dry slides were cleared in xylene for 4 min and covered with an anhydrous mounting medium. Pictures of sensorimotor cortex were taken on a Nikon DS-5Mc digital camera on a Nikon Eclipse 80i epifluorescence microscope.

### RNA Extraction From Sensorimotor Cortex Samples

The whole sensorimotor cortex was isolated in order to perform next generation sequencing (*n* = 3). Twelve hours/three days after injury, SHAM controls (P-15 12 h and 3 days, P-90 12 h and 3 days) and SCI mice (P-15 12 h and 3 days, P-90 12 h and 3 days) in triplicate experiments were sacrificed by cervical dislocation and brains were removed. We isolated the layer V of the sensorimotor cortices from 1 mm thick coronal sections obtained using a brain matrix, excluding as much as possible upper and lower layers and the subcortical white matter. The samples were individually collected and stored at −80°C. We extracted RNA as previously described (Valsecchi et al., 2015) using the mirVana extraction kit following supplier’s instruction (Life Technologies, Milan, Italy). The quality and quantity of RNA samples was checked by Nanodrop and the samples were stored at −80°C until sRNA library preparation. Only samples with a 260/280 and 260/230 ratio around 2.1 and 1.8, respectively were used.

### Small RNA Library Preparation and Next Generation Sequencing

Small RNA libraries were generated using HD adapters (Sorefan et al., 2012; Xu et al., 2015). HD adapters contain four degenerate nucleotides on the ligating ends of 5′ and 3′ Illumina adapters. HD adapters represent a pool of sequences rather than one fixed sequence, which increases the annealing efficiency between sRNAs and adapters. Increased annealing efficiency leads to a greater sRNA complexity in the libraries that are sequenced. Sequencing was performed on a HiSeq 2500 (Illumina).

### Bioinformatics

Raw FASTQ files were converted to FASTA format. Reads containing unassigned nucleotides were excluded. The 3′ adapter was trimmed by using perfect sequence similarity to the first 8 nt of the 3′ HiSeq 2500 adapter (TGGAATTC). The HD signatures (four assigned degenerate nucleotides at the ligating ends) of the reads were also trimmed. sRNAs were mapped full length with no mismatches to the mouse genome (GRCm38.p6) and then to latest set of mouse miRNA [miRBase (v22)] (Kozomara and Griffiths-Jones, 2014) using PatMaN (62). Normalisation and differential expression analysis was performed using DESeq2 (v1.2.10) (Love et al., 2014). Independent filtering was used to remove low-expressing miRNA (<5) in normalised counts. miRNAs were considered differentially expressed if they had a *p*-value < 0.05, <5% false discovery rate according to the Benjamini–Hochberg procedure and greater than log_2_ fold change >1.

### Quantitative PCR

miR-7b-3p, the miRNA with the highest sequencing score across three different groups, was selected for validation by quantitative PCR (qPCR). Total RNA was obtained from three SCI and three SHAM controls (sequencing samples) plus five SCI and five SHAM from the P-15 group. We also obtained RNA from three SCI and three SHAM controls (sequencing samples) plus two SCI and two SHAM controls for the P-90 group. We used the high capacity cDNA reverse transcription kit (Life Technologies) following supplier’s instructions. qPCR was performed with SYBR green core reagent kit and TaqMan assays (Life Technologies) on a Step-One 2000 PCR system. miRNA expression was analysed using RNAU6 as a housekeeping gene and *t*-test as statistic method. Samples were amplified simultaneously in triplicate in one assay run.

### Functional Analysis of miR-7b-3p Targets

Functional analysis of miR-7b-3p targets was performed following two different strategies. Based on the sequence homology of the seed region between miR-7a-2-3p (5′-CAACAAGUCCCAGUCUGCCACA-3′) and miR-7b-3p (5′-CAACAAGUCACAGCCAGCCUCA-3′) we selected a list of seven target genes already validated for miR-7a-2-3p by MiRWalk: *Zdhhc9*, *Wipf2*, *Cplx1*, *Basp1*, *Pfn2*, *Prkcb*, and *Snca*. These genes are known to be involved in neuronal differentiation. We screened using the miRWalk PTM the genes predicted to be targets of miR-7b-3p, selecting those genes recognised by six different databases. DAVID 6.7 was employed to obtain the enriched annotation terms of the listed genes. We selected 66 genes among the most interesting KEGG pathways (e.g., axon guidance, negative regulation of anoikis, regulation of actin cytoskeleton, and PI3K-Akt signalling pathway) with *p*-values ≤ 0.05.

### Analysis of miR-7b-3p Targets

The putative selected target genes of miR-7b-3p were validated by qPCR in the experimental groups that showed the highest miRNA level expression. The same total RNA extracted for miRNA validation was employed to check the expression of the seven target genes in P-15 3 days and P-90 3 days groups, the selected 66 genes in P-15 3 days and P-90 12 h groups. cDNA was synthesised using the high capacity cDNA reverse transcription kit following supplier’s instructions (Qiagen/Life Technologies). Two different qPCRs were performed: single analysis by SYBR green core reagent kit (Life Technologies, for the seven target genes) or custom plates by SYBR green technology (Bio-Rad for 66 selected genes) in a Step-One 2000 PCR system. Gene expression was analysed using *RS18* as a housekeeping gene. Samples were amplified simultaneously in triplicate in one assay run for the seven target list and pulled together (five SHAM controls vs five SCI; *t*-test as statistic tool) for the analysis of the 66 selected genes.

### Primary Cortical Neuron Cell Culture

In order to study miRNAs of interest *in vitro*, we isolated and cultured murine cortical neurons as previously described (Chiotto et al., 2019). Cells were obtained from murine C57BL/6J brain (*n* = 6 different embryos each dissection) at embryonic day 14.5 (E14.5). Cortices were dissected under the microscope and collected in 1× HBSS supplemented with 0.7% of HEPES and 1% of PS (Invitrogen-Gibco). Cells were enzymatically dissociated by trypsin-EDTA 0.05% (15 min at 37°C) and washed in the same HBSS dissection solution. We then added DNAse (1000U, Promega, Milan, Italy) to the dissection solution prior to mechanical dissociation by glass Pasteur pipette. Neurons were counted on a Burker chamber and plated at a density of 32,500 cells/cm^2^ on Poly-L-Lysine coated coverslips (0.1 mg/ml poly-L-lysine) in 1× MEM medium supplemented with 20% glucose, 1% of L-glutamine and 10% of horse serum (Invitrogen-Gibco) and incubated at 37°C, 5% CO_2_ and 95% humidity. After 4 h 1× MEM was replaced with Neurobasal supplemented with 2% of B27 and 1% of L-glutamine (Invitrogen-Gibco). Coverslips with paraffin dots were placed inverted on the cells to create a suitable environment for neuron differentiation. Neurons were cultured for 1–18 days.

### Neuro2a Cell Culture and Damage

Neuro2a (N2a) cells were grown in DMEM high glucose containing 5% FBS, 1% of PS and 1% of L-glutamine (Invitrogen-Gibco). In order to promote neurite extension, FBS concentration was decreased from 5 to 1% for 72 h before inducing the stress (Fukui et al., 2011). Cells were plated at a density of 32,000 cells/cm^2^ and were treated with 0.5 μM of hydrogen peroxide for 24 h as oxidative stress, as previously described (Fukui et al., 2011). The day after the cells were transfected (lipofectamine 2000, Invitrogen) with 5 nmol of miR-7b-3p mimic (Invitrogen) and after 72 h collected for RNA and protein extraction.

### Cell RNA Extraction and qPCR

To perform qPCR to measure the expression level of miR-7b-3p, cells were treated as follows: medium was removed and cells were incubated with trypsin-EDTA 0.05% at 37°C. Then the detached cells were centrifuged at 1,000 RPM for 5 min, washed with 1× PBS and pellet was stored at −80°C. RNA was extracted and quantified by Nanodrop as previously described for *in vivo* experiments. RNA purification was applied before performing the qPCR for miR-7b-3p using a solution of 1/10 volume of NH_4_OAC (0.5 M), 2.5 volume of cold 100% EtOH and 1 μl of glycogen (Invitrogen) for each tube. Samples were incubated at 80°C for 30 min and centrifuged at 12,000 *g* at 4°C for 20 min. The supernatant was removed, the pellet washed with 75% cold EtOH and centrifuged again at 12,000 *g* at 4°C for 5 min. The pellet was resuspended in DEPC water. RNA samples were stored at −80°C before qPCR. qPCR at 1, 7, and 18 days was conducted in triplicate. The qPCR for miR-7b-3p was performed as previously described for *in vivo* experiments. The expression values (7 and 18 days) were normalised to 1 day.

### Nucleofection

Cortical neurons were electroporated immediately after tissue dissociation and before plating using the rat neuron nucleofector kit (Amaxa, Switzerland). In brief, 500,000 cells were centrifuged for 5 min at 1,000 rpm. After that, supernatant was removed and neurons were resuspended in 100 μl of Nucleofector solution. Then, 5 nmol of miR-7b-3p mimic (Invitrogen) per 1 × 10^6^ cells or negative control (Invitrogen) were added to the suspension (Buller et al., 2010). Neurons were electroporated with the Amaxa programme O-003. Finally, neurons were plated on poly-L-lysinate coverslip at a final concentration of 32,500 cells/cm^2^.

### Immunocytochemistry

Three days after nucleofection, cortical neurons grown on coverslips were fixed with 4% PFA for 15 min and rinsed three times in 1× PBS. Permeabilisation was carried out with 0.1% TritonX-100/1× PBS for 5 min and non-specific binding sites were blocked by 5% BSA/PBS for 30 min. The following primary antibodies were incubated for 1 h: 1:200 monoclonal mouse anti-SMI-32 (Biolegend, SMI-32P San Diego, CA, United States), 1:1,000 monoclonal rabbit anti-βIII-tubulin III (Sigma-Aldrich, T8660, St Luois, MO, United States) and 1:400 monoclonal cleaved caspase 3 (9664, Cell Signaling). After washing in 1× PBS, primary antibodies were detected with anti-rabbit or anti-mouse cyanine 3-conjugated secondary antibodies, anti-rabbit Alexa-596 conjugated secondary antibody and Phalloidin TRITC (Sigma-Aldrich) 546 (1:1,000) for 30 min. Once mounted, samples were examined and images acquired using an Olympus Fluoview 300 confocal laser scanning microscope (CLSM). To check miR-7b-3p overexpression, qPCR was performed in triplicates on samples at 3 days after nucleofection.

### *In vitro* Morphometric Analysis and Evaluation of Apoptosis

The images acquired at CLSM were then analysed with ImageJ software. Five different parameters were measured, (i) axon length (*n* = 3), (ii) number of neurites (*n* = 3), (iii) area of the growth cone (*n* = 5), (iv) percentage of the growth cone shape (*n* = 5), and (v) stage of the neuronal development (*n* = 4). Differentiated cells were defined as those bearing at least one neurite longer than twice the cell body. For each experiment at least 30 axons/neurites were measured. The same cells were also analysed for the number of dendrites emerging from the cell body. Both measures were expressed as the total mean length for cell electroporated with negative control and miR-7b-3p mimic. The area of the growth cones was measured by tracing in ImageJ while the percentage of the growth cone shape was calculated as the ratio of one growth cone morphology (fork, hand, and stick) on the total number of growth cones. The classification of the stage of neuronal development (stage I, II, and III) was analysed as previously described (Takano et al., 2015). Here the percentage of neurons at each stage was calculated on the total number of neurons analysed.

As concerns, the analysis of apoptotic signs/markers, the morphology of the nuclei and the expression of cleaved caspase 3 were analysed. A number >30 of neurons were analysed in sham and mimic conditions and results presented as the percentage of apoptotic nuclei or cleaved caspase 3-positive cells over the total number of nuclei/cells.

### Western Blotting

After 72 h from the transfection, both primary cortical neurons and N2a were collected (*n* = 3), the lysate centrifuged and the protein content measured by the BCA protein assay kit (Invitrogen). Protein extracts (15 μg) were separated on 10% SDS-poly-acrylamide gels and transferred to PVDF membranes. After washing in Tris–HCl-buffered saline 0.2% Tween-20 (TBST), the membranes were blocked for 1 h at RT in TBST with 5% milk and eventually incubated with the primary antibody (1:2,000 α-actin, A2066; 0.4 μg/ml Wipf2-HPA024467, Sigma-Aldrich) overnight at 4°C. Goat anti-rabbit IgG HRP (Invitrogen) was used as secondary antibody (1:2,000) for 1h at RT. The protein bands were detected with a western light chemiluminescence detection system (ECL, GE Healthcare Bio-Sciences AB) and photographed in an ImageQuant LAS 4,000 mini (GE Healthcare Bio-Sciences AB). The images were analysed by ImageJ, and cropped for presentation.

### Statistical Analysis

Data were evaluated as mean ± standard error of mean (SEM). Statistical analysis was performed using Prism 6.0 (GraphPad, San Diego, CA, United States). For the histological analysis the percentage of GFAP/IBA1 positive area was quantified using ImageJ and analysed with a non-parametric *t*-test (Mann–Whitney). Values *p* ≤ 0.05 were considered statistically significant. In the sRNA library preparation and sequencing experiments, the empirical differential expression analysis was confirmed by parametric (*t*-tests) and non-parametric (Mann–Whiney U) tests. For the statistical tests we considered *p* < 0.05 as statistically significant. miRNAs were considered differentially expressed if there was a >0.5 log_2_ fold change between controls and treatments, i.e., more than 1.5-fold change. To validate miR-7b-3p and its targets by qPCR, changes in miRNA/gene levels were detected as the difference in threshold cycle (ΔCT) between the target gene and the housekeeping gene. The results were analysed by Mann–Whitney test and *t*-test and presented as normalised values between SHAM controls and SCI groups. In the nucleofection experiments, the statistical analysis was performed by a non-parametric *t*-test (Mann–Whitney). For the morphometric analysis *t*-test was employed for the axon length, number of neurites and growth cone analysis, the χ^2^ test was employed for the neuron stage analysis and values *p* ≤ 0.05 were considered statistically significant. The count of apoptotic nuclei and the expression of cleaved caspase 3 was analysed by *t*-test. In the western blotting the protein bands were analysed by *t*-test and the data presented as relative protein level.

## Results

### SCI Triggers Neuroinflammation but Not Evident Neuronal Degeneration at Cortical Level

C57BL/6J male mice underwent a complete spinal cord transection at the cervical level 6 (C6) by a syringe needle. To characterise this injury model, we analysed the inflammatory reaction and the presence of degenerating cells in sensorimotor cortex 12 h and 3 days post SCI in both young (P15) and adult cohorts (P90). The level of microglia activation (IBA-1 positivity) increased in all the SCI groups (*t*-test, *t*-statistic, *p* < 0.0001) also at the cortical level (Supplementary Figure 1). In adult mice no signs of astrogliosis (GFAP reactivity) were observed in the cortex (data not shown). Altogether these results suggest that the spinal cord lesion can trigger an inflammatory response also at cortical level. We did not detect degenerating cells by FJC staining in the sensorimotor cortex, whereas degenerating cells were markedly visible in the spinal cord of SCI mice (Supplementary Figure 2). These results seem to indicate that the lesion model does not activate a cell death pathway at the cortical level.

### miR-7b-3p Is the Major Upregulated miRNA in SCI-Sequencing Profile

**FIGURE 1:**
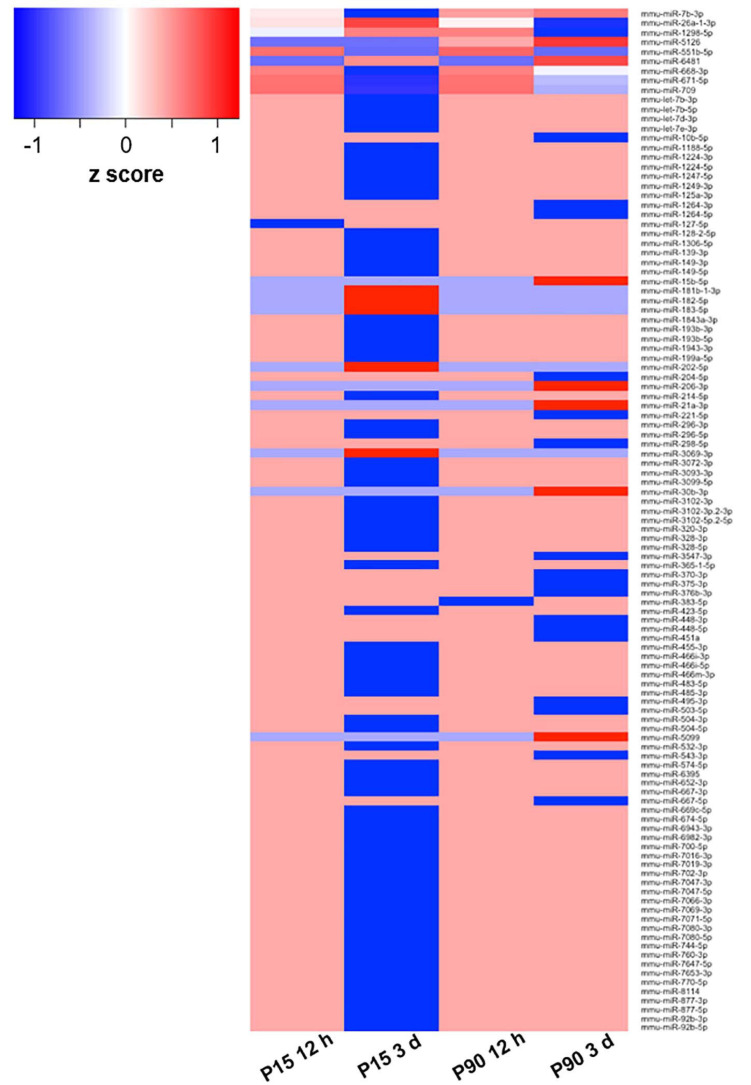
Deregulated miRNA after SCI. Heat map based on log_2_ fold change values of 110 DE miRNAs in at least one of the comparisons between cohorts (P15 12 h, P15 3 days, P90 12 h, P90 3 days) plus corresponding controls. The *z*-score represents the deviation from the mean by standard deviation units; *n* = 3 for each experimental group.

To evaluate miRNA differential expression after SCI, a complete transection of the spinal cord was performed in P-15 and P-90 mice. sRNA sequencing was then performed on isolated layer V sensorimotor cortex tissue, sampled at 12 h and 3 days after SCI (Figure 1).

To select a candidate miRNA for further investigation, we looked for correlations/relationships between differentially expressed (DE) miRNAs and groups. Three miRNAs were upregulated (miR-551b-5p, miR-6481, and miR-5126), while four were downregulated (miR-1298-5p, miR-668-3p, miR-671-5p, and miR-709) and shared between two groups. Instead miR-7b-3p and miR-26a-1-3p were both upregulated and shared among three groups (P15-12 h, P90-12 h, and P90-3days, and P15-12 h, P15-3 days, and P90-12 h, respectively). These two miRNAs were found in both young and adult mice with miR-7b-3p showing the highest fold change (log_2_ fold change) when compared to controls (Table 1). This data suggested that miR-7b-3p could perform gene regulatory functions related to SCI independent of animal age and may have stronger biological/clinical relevance. To better understand the role of miR-7b-3p, we screened the literature using PubMed and miRpub, a specific database collating miRNA-related articles. We focused on functions related to neurite/axon outgrowth during development, following a lesion and/or specifically related to SCI. Although some evidence had been reported in the literature for miR-7b-3p, there were no validated target genes reported in miRWalk (Table 1).

**TABLE 1:** miR-7b-3p general information.

	**Log_2_ fold change**	**Experimental group(s)**	**Functions**	**References**	**miRWalk validated target(s)**
miR-7b-3p	1.56 1.99 2.17	P-15-12 h P-90-12 h P-90-3 days	• Cerebral cortex development • Neurite outgrowth • Synaptic formation • OL specification • PD, schizophrenia, SCI pathogenesis and ischemia	•Chen et al., 2010 •Pollock et al., 2014 •Liu et al., 2012 •Zhao et al., 2012 •McMillan et al., 2017 •Beveridge and Cairns, 2012 •Liu et al., 2009 •Liu et al., 2018	No validated targets

*miRNA functional analysis by miRpub, PubMed, and miRWalk. In the second column, log_2_ fold change is reported (*n* = 3).*

### q-PCR Confirmed the Upregulation of miR-7b-3p in the Cortex of SCI

**FIGURE 2:**
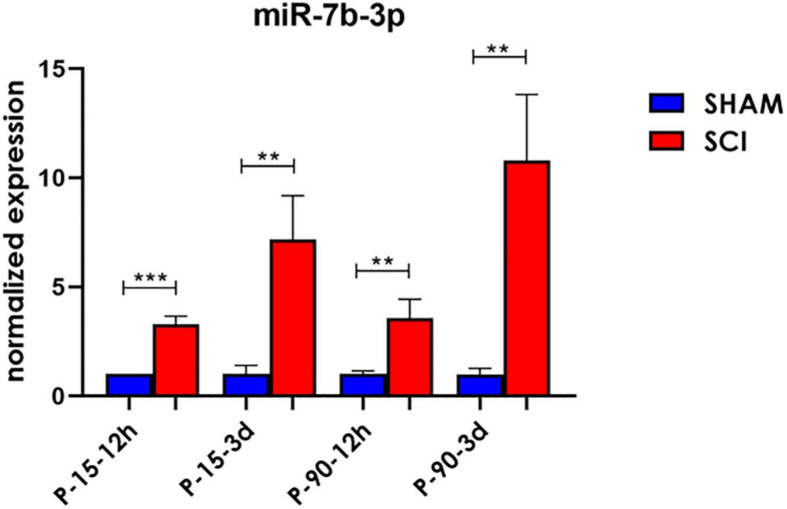
miR-7b-3p upregulation following SCI. miR-7b-3p upregulation in all SCI groups was confirmed by RT-PCR. The graph expresses miRNA normalised relative expression values ± SEM (Mann–Whitney ***p < 0.001; **p < 0.01; *n* = 8 for each group).

To confirm the sequencing results, miR-7b-3p was further analysed by qPCR to confirm its DE in SCI compared to SHAM controls. A significant upregulated fold change of 2.28 ± 0.37, 1.57 ± 0.85, and 9.8 ± 3.01 times was observed for miR-7b-3p in SCI P-15 12 h, P-90 12 h, and P-90 3 days, respectively, compared to controls (*t*-test, *t*-statistic, SHAM vs SCI *p* < 0.0006 in P-15 12 h; *p* < 0.007 in P-90 12 h; *p* < 0.007 in P-90 3 days). We also found that miR-7b-3p expression level was 6.16 ± 2.02 times higher in SCI P-15 3 days mice compared to SHAM controls (Figure 2; *t*-test, *t*-statistic, SHAM vs SCI *p* < 0.004). The expression of miR-7b-3p increased over time between 12 h and 3 days in both animal age groups. This data suggested that miR-7b-3p could exert a specific role connected to SCI independently of animal age and time after lesion induction.

### miR-7b-3p Targets Genes Essential for Neurite Outgrowth

**FIGURE 3:**
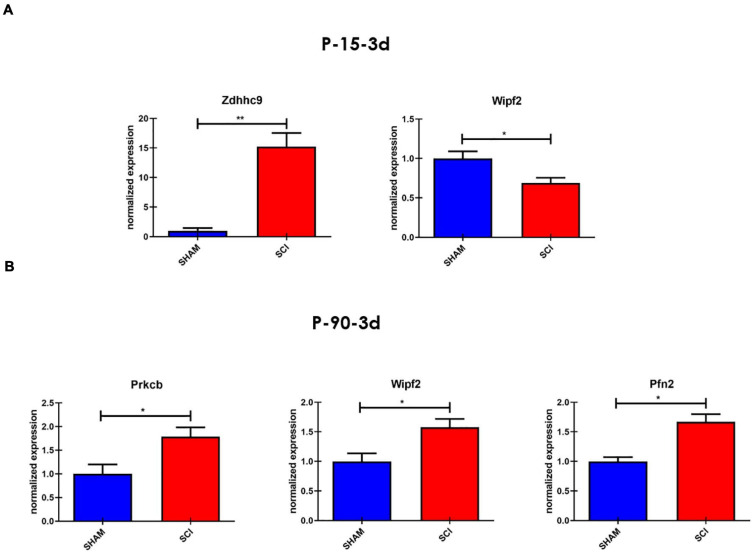
The targets of miR-7b-3p. **(A)** Two/seven genes were found to be up and downregulated in P15-3d SCI group, while three/four resulted upregulated in P-90-3 days SCI **(B)**. The graphs express normalised relative expression values (RT-PCR) ± SEM (*t*-test **p ≤ 0.002; *p < 0.01; *n* = 5 for each group).

To better investigate the role of miR-7b-3p in regulating the axon guidance process, we validated several miR-7b-3p target genes by qPCR. Since the seed sequence is shared between miR-7a-2-3p and miR-7b-3p, we first analysed the seven genes already known to be targets of the first miRNA and directly linked to different neuronal processes (Supplementary Table 1). As shown in Figure 3, *Wipf2* expression (*t*-test, *t*-statistic, *p* = 0.02) decreased in SCI P-15 3 days animals compared to controls (Figure 3A); *Zdhhc9* expression (*t*-test, *t*-statistic, *p* = 0.002) was increased in SCI P-15 3 days (Figure 3A); *Prkcb* (*t*-test, *t*-statistic, *p* = 0.04), *Wipf2* (*t*-test, *t*-statistic, *p* = 0.02), and *Pfn2* (*t*-test, *t*-statistic, *p* = 0.01) were increased in P-90 3 days SCI mice compared to SHAM controls (SMAH vs SCI). Since *Wipf2* is known to be essential for neurite outgrowth/extension, this result implicates its possibility as a direct miR-7b-3p target. Considering that *Zdhhc9*, *Prkcb*, and *Pfn2* are linked to axon growth (Supplementary Table 1), their upregulation in SCI could represent an indirect link between miR-7b-3p and their relative expression.

**FIGURE 4:**
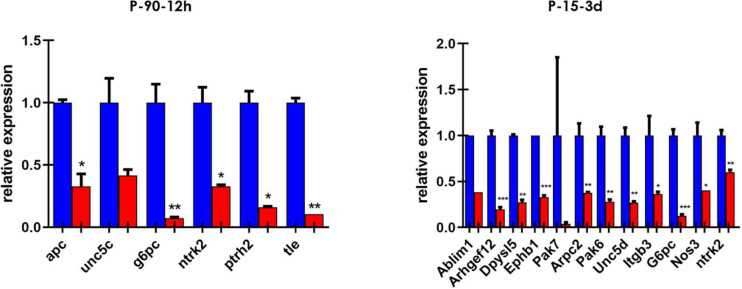
Custom card of putative miR-7b-3p targets. Downregulated genes in P-90-12 h and P-15-3 days experimental groups. Data are shown as normalised relative expression values of technical replicates (RT-PCR, *n* = 3 technical replicates; *p ≤ 0.04, **p ≤ 0.09, ***p ≤ 0.0006).

We then screened a selected list of genes predicted to be targets of miR-7b-3p by qPCR custom plates in two of the experimental groups. We chose 66 genes belonging to the following KEGG pathways: axon guidance, negative regulation of anoikis, regulation of actin cytoskeleton, PI3K-Akt signalling pathway. Among all the genes analysed, 16 were downregulated in the cortex of SCI mice and among them, two (*G6pc* and *Ntrk2*) were shared between the two groups (Figure 4). Only some genes have been experimentally demonstrated as key components of the KEGG pathways. The function of some genes such as *G6pc*, *Ptrh2*, and *Itgb3* in this context remains unclear (see Supplementary Table 2).

### miR-7b-3p Is Not Responsible of Neurite Outgrowth During *in vitro* Cortical Neuron Development

**FIGURE 5:**
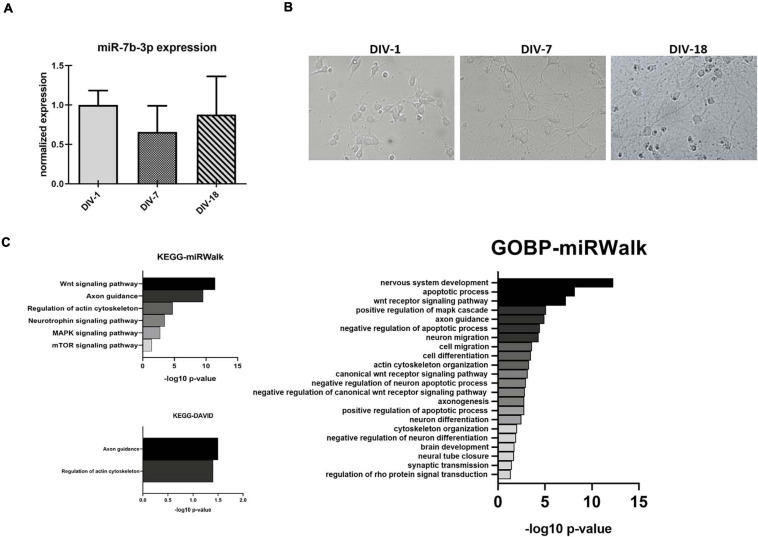
miR-7b-3p expression and functional analysis in primary cortical neurons. **(A)** Developing *in vitro* cortical neurons, DIV-1 undifferentiated neuron stage, DIV-7 dendritogenesis stage, and DIV-18 mature neuron stage; **(B)** miR-7b-3p expression at DIV-1-7-18 presented as normalised relative expression value compared to DIV-1; **(C)** enriched annotation terms with a significant *p*-value analysed by miRWalk and DAVID 6.7 (*n* = 3; values expressed as –log_10_ *p*-value).

In order to better understand the role of miR-7b-3p, we evaluated whether this miRNA could be related to the axon growth during cortical neuron development, a function that could be exploited as potential SCI target (Hilton and Bradke, 2017). We used an *in vitro* system to mimic three different time points, 1, 7, and 18 days corresponding to the undifferentiated neuron stage, dendritogenesis beginning stage, and mature neuron stage, respectively (Figure 5A). miR-7b-3p expression was constant during neuron development with slight oscillations among the time groups possibly suggesting its expression change reactivation only after SCI (Figure 5A).

**FIGURE 6:**
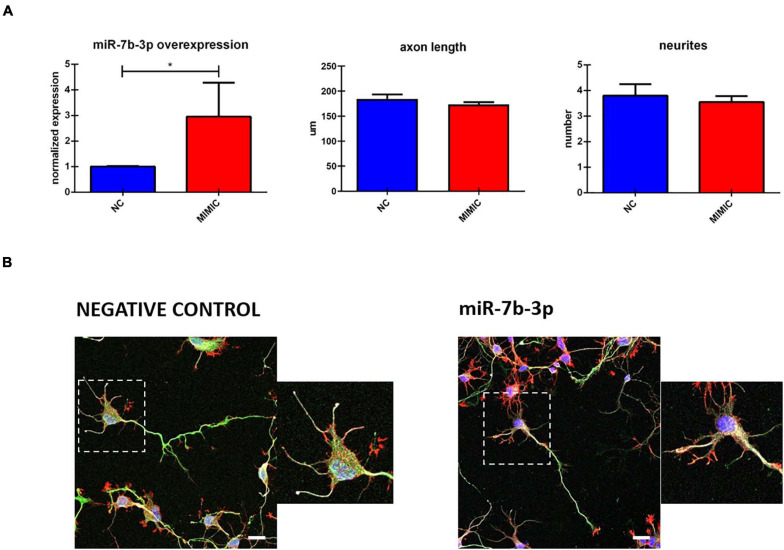
miR-7b-3p overexpression and its role in neurite outgrowth. **(A)** miR-7b-3p overexpression and measurement of axon length (*n* = 3) and number of dendrites (*n* = 3). **(B)** Comparable axon length and number of dendrites in NC (negative control) and mimic-7b-3p electroporated cells. Scale bars: **(B)**, 20 μm. The graphs express miRNA normalised relative expression value, μm for axon length and number for neurites ± SEM (*t*-test *p 0.02).

To further analyse miR-7b-3p function during development, we studied *in vitro* the morphological phenotype of cortical neurons in which we overexpressed this miRNA. Cells transfected with miR-7b-3p mimic showed a significant upregulation of 3.05 (*t*-test, *t*-statistic, *p* 0.02) times compared to the negative control transfected cells (Figure 6A). However, miR-7b-3p overexpression did not change the axon length or the number of neurites (Figures 6A,B). These results suggest that miR-7b-3p is not involved in neurite or axon growth during the cortical development.

### miR-7b-3p Maintains the Cells in a More Plastic Stage

Since no validated targets are known for this miRNA, we performed a functional analysis based on putative targets predicted by (i) miRWalk algorithm or (ii) miRWalk intersection among 12 different databases. We considered only those genes predicted by at least 6 databases for DAVID 6.7. Different KEGG and GOBP neuronal related functions (mainly connected to axon cytoskeleton, neural development, and apoptosis) showed a significant *p*-value (Bonferroni *post hoc* test, *p* ≤ 0.03, Figure 5C). Only two KEGG neuronal functions (axon guidance and regulation of actin cytoskeleton) reached statistical significance (Figure 5C).

**FIGURE 7:**
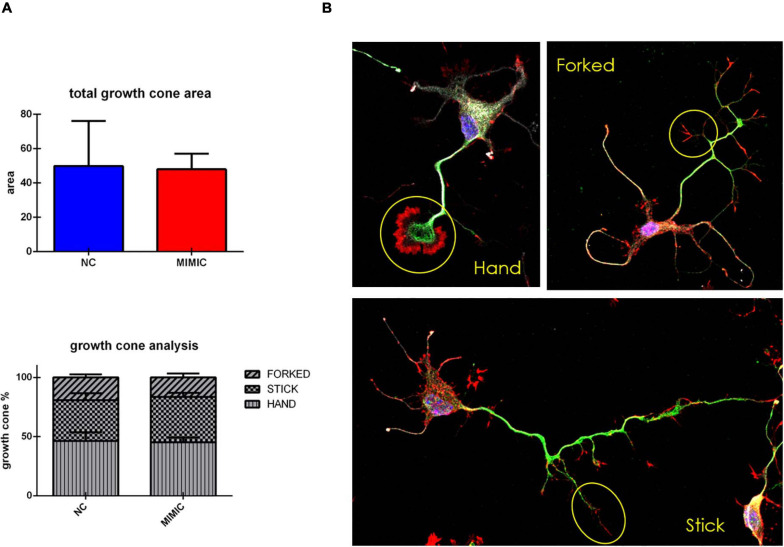
Growth cone analysis in cortical neurons overexpressing miR-7b-3p. **(A)** Measurement of the growth cone area and the percentage of “hand,” “forked,” and “stick” growth cone shape in mimic transfected cells. **(B)** “hand,” “forked,” and “stick” growth cone shape analysed in cortical neurons with SMI-32 (neurofilament in white), βIII-tubulin (cytoskeleton in green), phalloidin (actin in red). Scale bar: 20 μm. The graphs express the area and the percentage of each type of growth cone/total growth cone number ± SEM (*n* = 5; *t*-test n.s.).

On the basis of these results, we explored the effect of miR-7b-3p overexpression on the growth cone area and shape of cortical neurons. However, the total growth cone area was not affected by the transfection of miR-7b-3p mimic (Figure 7A). Similarly, the analysis of the ratio of the growth cone shape (forked, stick, and hand) on the total number of growth cone (expressed in percentage) did not reveal any significant change overexpressing the miRNA (Figures 7A,B).

**FIGURE 8:**
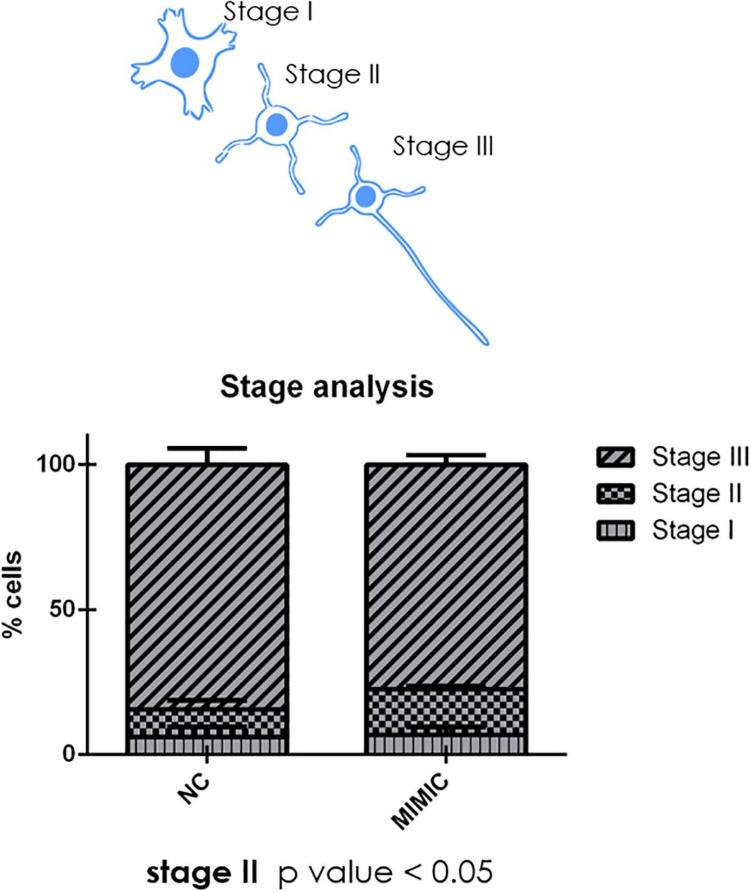
moiR-7b-3p increases the number of neurons in stage II. The number of stage I, II, and III neurons is expressed as a percentage on the total number of neurons analysed. An increased stage II number of neurons is shown (*n* = 4; *t*-test *p ≤ 0.05 for stage II).

During development cortical neurons switch from a round shape (stage I) to a ramified middle stage (stage II) and eventually reach the stage III in which the longest branch will become the axon of the neuron (Figure 8). We analysed the percentage of cells at each stage after mimic transfection. Interestingly, miR-7b-3p overexpression significantly increased the percentage of neurons at stage II (χ^2^ test, *p* = 0.04) (Figure 8). These results suggest a role of miR-7b-3p in maintaining the cells in a more immature and, likely plastic, neuronal developmental phase.

### miR-7b-3p Reduces Apoptosis in Primary Cortical Neurons

**FIGURE 9:**
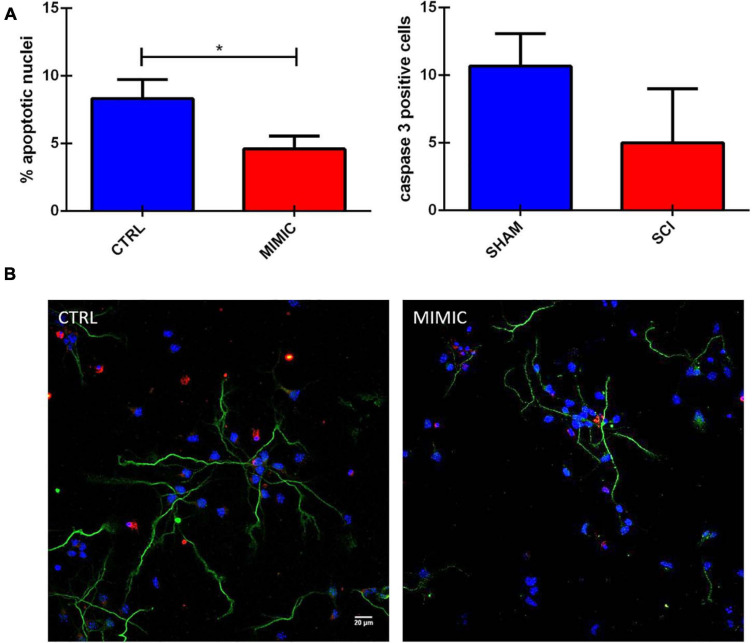
miR-7b-3p reduces the number of apoptotic neurons *in vitro*. **(A)** Analysis of the percentage of apoptotic nuclei (% values ± SEM, *t*-test, *p = 0.03) and cleaved caspase 3 in mimic overexpressing cells. **(B)** Cleaved caspase 3 expression (red), SMI-32 (green), DAPI (blue) in CTRL and MIMIC cortical neurons.

The functional analysis based on putative targets of miR-7b-3p also included the class of genes related to apoptosis. We then explored the effect of the overexpression of the miR-7b-3p in cortical neurons, evaluating the morphology of the nuclei and the expression of cleaved caspase 3 (a well know apoptotic marker; Crowley and Waterhouse, 2016). The overexpression of miR-7b-3p resulted in a significant lower percentage of apoptotic nuclei (Figure 9, *t*-test, *t*-statistic, *p* = 0.03) and a reduced expression of cleaved caspase 3 compared to the control cells. This result indicates that miR-7b-3p can counteract the apoptotic mechanisms and exert a neuroprotective effect.

### *In vitro* miR-7b-3p Overexpression Reduces Wipf2 Expression in Damaged but Not in Healthy Neurons

**FIGURE 10:**
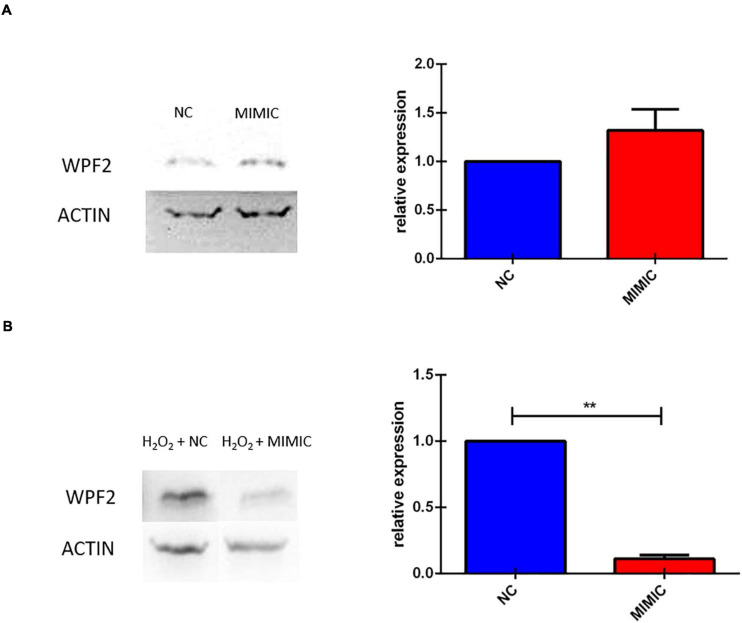
miR-7b-3p overexpression *in vitro* reduces Wipf2 protein level upon oxidative stress condition. **(A)** After miR-7b-3p mimic transfection, the relative expression of Wipf2 protein was quantified both in primary cortical neurons in healthy state and **(B)** in N2a cells undergone oxidative stress condition. Data are shown as normalized relative expression values (*n* = 3, **p ≤ 0.001).

We finally investigated the effect of miR-7b-3p overexpression on Wipf2 expression, one of the predicted target genes whose transcript was downregulated in P-15-3d mice (Figure 3). In healthy primary cortical neurons, the miRNA overexpression does not affect the Wipf2 protein expression, as shown in Figure 10A. However, N2a cells overexpressing miR-7b-3p (Supplementary Figure 3) upon an oxidative stress (H_2_O_2_ administration, mimicking the SCI environment), exhibited a significant decreased expression of Wipf2 (Figure 10B). This result confirms *Wipf2* as a direct target of miR-7b-3p, but only in damage conditions. Therefore, miR-7b-3p can downregulate the expression of neurite growth/regeneration related genes (as *Wipf2*) and, once again, this supports the hypothesis that miR-7b-3p can maintain the cells in a more immature state, possibly at the expense of the axon growth.

## Discussion

We investigated the miRNA profile in SCI in order to identify specific miRNAs involved in regeneration/plasticity. We performed next generation sequencing of the layer V sensorimotor cortex in both young and adult mice to evaluate miRNA dysregulation 12 h and 3 days after SCI. The only miRNA significantly upregulated in all of the evaluated conditions was miR-7b-3p, whose function in spinal cord lesion was the focus of this work.

Several studies analysed the global profiling of miRNA expression after SCI obtaining differential expression for a great number of sRNAs (Liu et al., 2009). Some miRNAs acting on different pathways promote functional recovery. For instance, miR-133b is an important determinant for axon regrowth and functional recovery by reducing RhoA protein in an adult zebrafish SCI model (Yu et al., 2011). miR-124 has been demonstrated to control at least four different pathways (PI3K/AKT, REST, and Rho1) all related to axon regrowth after injury (Ghibaudi et al., 2017). Despite the growing number of miRNAs connected to SCI condition, we still lack a complete comprehension of this phenomenon.

To identify a wider collection of miRNAs overexpressed/downregulated following SCI, we performed sRNA library construction with a recently improved technique (Sorefan et al., 2012; Xu et al., 2015). This method employs HD adapters that contain degenerate nucleotides at their ligating ends, allowing for the formation of more stable secondary structures between miRNAs and adapters prior to sequencing. Using this approach, sequencing bias in biological samples can be reduced and the representation of low abundance miRNAs increases leading to the identification of previously unknown miRNAs. For all the groups tested, sequencing generated a list of miRNAs potentially dysregulated in SCI cortex compared to SHAM control groups. Some miRNAs such as miR-7b-3p were reported more than once in the same group (Table 1). This repetition is due to the fact that slightly different sequences have been attributed to the same miRNA name. We chose the sequence with the most abundant number of reads as the most valid, such as in the case of miR-7b-3p.

MicroRNAs act in a network system in which different members can converge on the same transcripts (targets), on the same pathways or on the same functions (Barca-Mayo and De Pietri Tonelli, 2014; Ghibaudi et al., 2017). miRNet analysis did not reveal any significant interaction among the dysregulated miRNAs within each group or shared among the groups analysed (P-15 12 h and 3 days; P-90 12 h and 3 days). A list of seven miRNAs emerged as potentially related to axon growth, plasticity and regeneration pathways possibly reactivated after SCI. Consistent with this hypothesis, among the miRNAs predicted to be up/downregulated, only miR-7b-3p upregulation was confirmed by qPCR in three SCI groups (P-15 12 h and P-90 12 h, 3 days). miR-7b-3p belongs to the evolutionary conserved miR-7 family implicated in the normal function of different organs (pancreas, heart, and skin) with a particular enrichment profile in brain (Horsham et al., 2015). Indeed, specific RNA binding proteins and transcription factors (hnRNP and Yan) ensure miR-7 expression in neuronal cells and a refined control of cell differentiation through a negative feedback loop mechanism (Arora et al., 2013; Choudhury et al., 2013). Consistently with the functional analysis of the putative predicted targets of miR-7b-3p, we focused on three gene classes: axon cytoskeleton, neural development, and apoptosis.

The role of the miR-7 family in the CNS is heterogeneous ranging from neuroprotective effect to alleviation of the inflammatory reaction in Parkinson’s disease (PD) and stroke, respectively (McMillan et al., 2017; Zhang et al., 2018; Xu H. et al., 2019). In SCI miR-7 function is poorly understood. The literature often refers to other members of the miR-7 family that do not share the same seed region with the miR-7b-3p here analysed. We screened the validated targets involved in neuronal processes of miR-7a-2-3p that has the same seed region of miR-7b-3p. Only four genes were significantly modulated in SCI conditions. The first gene, *Wipf2*, is a gene required for neurite branching and outgrowth, lamellipodia formation and migration (Banzai et al., 2000; Suetsugu et al., 2001; Kitamura et al., 2003; Kakimoto et al., 2004; Takenawa, 2005; Xiao et al., 2008) whose level of expression decreased in the P-15 3 days upon miR-7b-3p upregulation after SCI. This result suggests *Wipf2* as a direct target of the miR-7b-3p, that unexpectedly seems to negatively influencing the axonal regrowth after SCI. This was further demonstrated by our *in vitro* experiments (Figure 10B). On the other hand, *Zdhhc9*, *Prkcb*, and *Pfn2* were found upregulated in P-15 3 days and P-90 3 days groups. Among their different functions, all these genes are required in the regulation of actin stability (Da Silva et al., 2003), axon branching (Han et al., 2015; Holland and Thomas, 2017) and neural maturation and differentiation (Guo et al., 2012; Kaur et al., 2014), but their role after injury has not been postulated. Consistently with their known functions and the increased level of expression we found after SCI, their reactivation can be interpreted as an indirect effect of miR-7b-3p overexpression. This could therefore indicate the presence of secondary elements controlled by miR-7b-3p that lead to the reactivation of specific neuronal pathways.

We extended the analysis to those genes predicted by miRWalk as direct targets of miR-7b-3p, finding that 16 genes were downregulated in the SCI cortex of P-15 3 days and P-90 12 h. Only 2 of these 16 genes were the genes shared between the young and adult group, *G6pc* and *Ntrk2*. While *G6pc* role still remain unclear in this specific context (Supplementary Table 2), *Ntrk2* is known to control neurogenesis and axonal sprouting. miR-7b-3p overexpression decreases the level of 14 other genes in the P-15 3 days group (Supplementary Table 2). These genes are all involved in developmental axon guidance, neurite outgrowth and neurogenesis processes at different extents. The fact that some of them, including *Arhgef12*, *Ephb1*, and *Pak6*, have been already described to be upregulated in SCI can be ascribed to the different injury models employed and to the tissue in which the analysis has been conducted. Most of the SCI experiments are focused on the spinal injured tissue, while we shifted the observation at the cortical level so introducing a different perspective and revealing new potential targets.

Since it is known that the injured adult CNS re-expresses genes and activates pathways that are observed during neuronal development (Emery et al., 2003), we explored the function of this miRNA while this process is occurring. The expression of miR-7b-3p was constant during the *in vitro* neuronal developmental steps (Figure 5), as well as its overexpression in primary cortical neurons did not affect neither axon length nor the number of neurites nor the growth cone area or the percentage of different type of growth cones (Figures 6, 7). Conversely, the analysis of the neuronal stages showed that miR-7b-3p mimic increased the percentage of cells at stage II, a ramified middle stage (Figure 8), representing an intermediate phase of neuronal development where the cell must still determine which neurite will become the axon. This result suggests that, eventually also in case of SCI, increased level of miR-7b-3p could make the damaged neurons more plastic, in an attempt of the cell in refining the growth of neurites. This mechanism is part of the sprouting process required for a proper regeneration after a lesion and known to partially involve the reactivation of specific developmental pathways (Hilton and Bradke, 2017).

Altogether, these results can be actually explained by a complex and subtle role of miR-7b-3p. Indeed, the regrowth of a severed axon is a spatially and temporally controlled mechanism that needs to be timely regulated in order to find the most appropriate environmental conditions (Fawcett, 2020). In this context, it is possible that miR-7b-3p downregulates genes promoting axon regeneration, maintaining the neurons in a more plastic phase (resembling the *in vitro* stage II) redefining the neurite will be the axon.

The conservation of genes involved in the regeneration/plasticity process could also be the key to explain the absence of differences we observed between the young and the adult SCI groups. Although at P-90 the nervous system is considered completely mature and less plastic compared to P-15 (when neuronal networks are still defining), the molecular pathways promoting the functional recovery after SCI involve neuronal development pathways (such as *Wnt* pathway) that are highly evolutionary conserved (Herman et al., 2018; Squair et al., 2018).

Moreover, interestingly, our data suggest another role of miR-7b-3p, linked to neuroprotection. It has been demonstrated that miR-7 silences pro-apoptotic genes (Pollock et al., 2014), a functional class that we also observed to be enriched in miR-7b-3p functional analysis (Figure 5C), and protects motor neuron *in vitro* (Chakrabarti et al., 2014). It has been recently shown that miR-7 (member of the same family) exerts a neuroprotective effect in PD directly acting on α-synuclein and reducing the apoptotic mechanism (Je and Kim, 2017; Tarale et al., 2018; Salama et al., 2020). These data are in line with our results showing that miR-7b-3p overexpression reduces the percentage of apoptotic nuclei and the expression level of cleaved caspase 3 (Figure 9).

Overall, we can hypothesize an intriguing dual role of miR-7b-3p in: (i) the induction of a more plastic neuronal condition/phase, possibly at the expense of the axon regeneration (as also demonstrated by our *in vitro* experiments), (ii) the neuroprotective role exerted through the inhibition of the apoptotic cascade. Overall, our findings suggest that increasing the miR-7b-3p levels in case of SCI could reactivate in adult neurons silenced developmental programmes, supporting at the same time the survival of the axotomised neurons.

Lastly, a final consideration on our results is needed to explain the few differences we observed in *our* experiments: (i) the miRNA sequencing profile we analysed derived from a mixed cell population also containing astrocytes, microglia and interneurons whose contribution cannot be totally excluded; (ii) the primary cortical neuron model we employed does not fully recapitulate the pathophysiological phases occurring after SCI, thus probably underestimating some possible additional effects.

## Conclusion

MicroRNAs emerged as new alternative targets in SCI as they are a potent inner regulatory system of gene expression that can be manipulated. We laid the groundwork for the future investigation of the potential roles of miR-7b-3p, whose action can represent an intriguing therapeutic target. Although these experiments need further confirmation, miR-7b-3p could be linked to the modulation of plasticity-related genes and to a neuroprotective function, both part of the same mechanism to support the survival and regeneration after SCI. Manipulation of one single miRNA cannot be considered the most effective therapeutic strategy, but our results allowed to better understand miRNA mechanisms of action and add new elements to the miRNA complex network in SCI. A more complete list of long non-coding RNA involved in the regrowth programme will help to design integrative approaches with a stable and successful therapeutic value.

## Data Availability Statement

All data supporting the findings of this study are available within the article and Supplementary Material or from the corresponding author on request. Raw sequencing files are available at Gene Expression Omnibus (www.ncbi.nlm.nih.gov/geo) under the accession GSE89517.

## Ethics Statement

The animal study was reviewed and approved by the European Activities Communities Council Directive of 86/609/EEC 1986 and University of Turin institutional guidelines on animal welfare.

## Author Contributions

MG and AV: study and experiment design. MG, MB, DG, ES, GB, and SP: experiments. AS: bioinformatics. MG, DG, AS, TD, and AV: data analysis. MG, MB, and AV: manuscript draft. All authors contributed to the article and approved the submitted version.

## Conflict of Interest

The authors declare that the research was conducted in the absence of any commercial or financial relationships that could be construed as a potential conflict of interest.
