# Supplementary material for: miR-7b-3p Exerts a Dual Role After Spinal Cord Injury, by Supporting Plasticity and Neuroprotection at Cortical Level
Source: Front Mol Biosci. 2021 Mar 31;8:618869. doi: 10.3389/fmolb.2021.618869 (PMC8044879; doi:10.3389/fmolb.2021.618869)
Supplement: Supplementary file 1 [file Data_Sheet_1.pdf]

SUPPLEMENTARY MATERIAL

Figure S1. Inflammatory process occurring at the sensory motor cortex level: (A-B) the P-15 SCI group present an increased macrophage activation both at 12h (16.82%) and 3d (19.16%) after the lesion, compared to the sham group (6.69% at 12h and 6.79% at 3d). Moreover, at both time points, the morphology of macrophages appear different in comparison to controls: P-15 SCI mice are predominantly characterized by both ramified and amoeboid cells (A-a’’, B-b’’insets). (C-D) The same pattern of macrophage activation is also present in both adult groups (C-c’’, D-d’’), although the density of IBA-1-immunopositive profiles is unchanged compared to the sham animals. Data are shown as percentage of positive area of IBA-1 ± SEM. **** p = 0.0001. Scale bars: A,B,C,D 50 µ, a’/’’, b’/’’, c’/’’, d’/’’ insets 10µm.

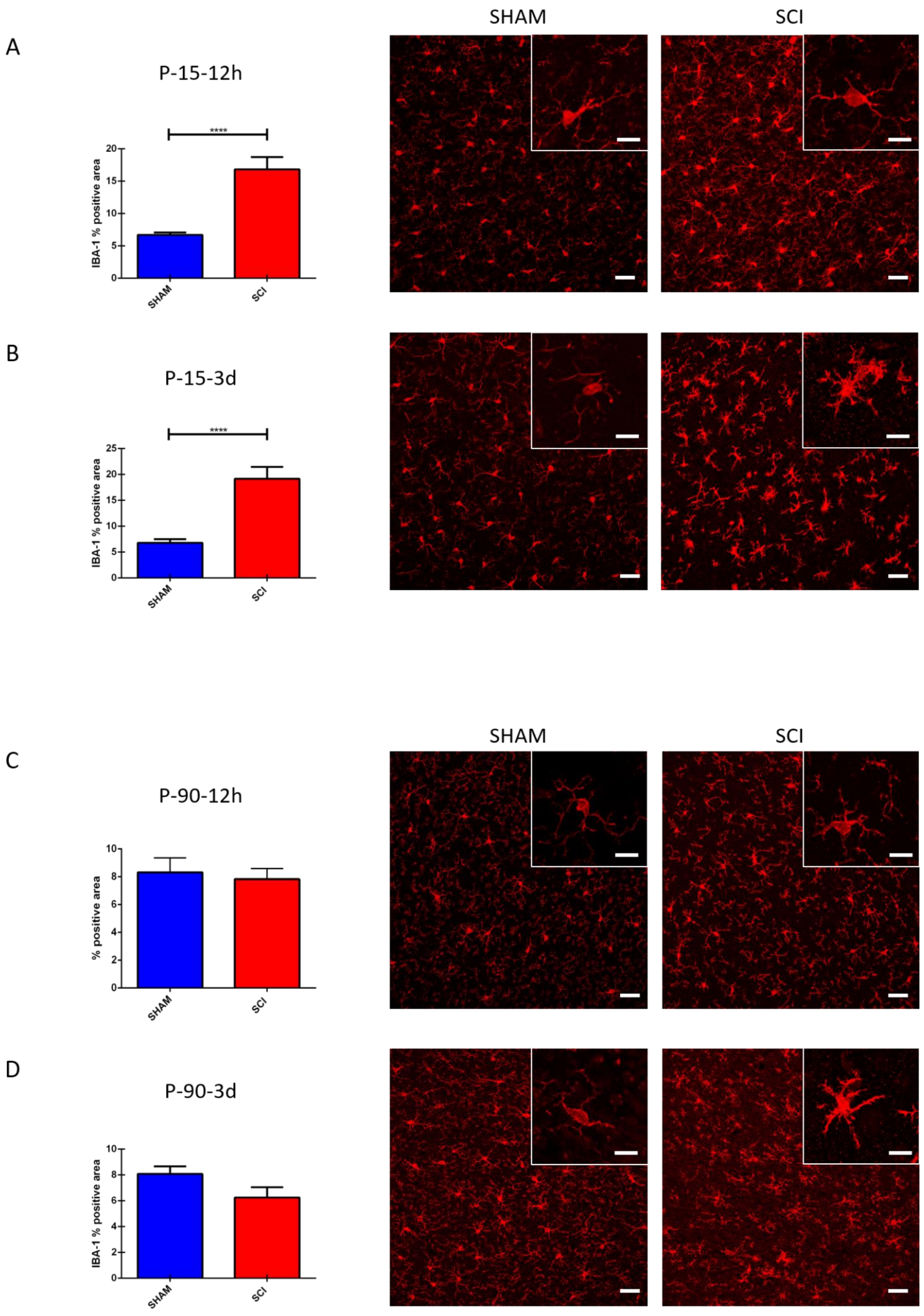
SHAM
SCI
A
P-15-12h
****
IBA-1 % positive area
0
5
10
15
20
SHAM
SCI
B
P-15-3d
****
IBA-1 % positive area
0
5
10
15
20
25
SHAM
SCI
SHAM
SCI
C
P-90-12h
% positive area
0
2
4
6
8
10
SHAM
SCI
D
P-90-3d
IBA-1 % positive area
0
2
4
6
8
10
SHAM
SCI

Figure S2. FJC staining in motor cortex (MC) and spinal cord (SC) of SHAM and SCI mice. Both P-15 (A) and P-90 (B) SCI groups do not present any degenerating cells into the motor cortex, whereas they are visible in the spinal cord of SCI mice at the lesion site (green labelling in SC-SCI). As expected, no signs of cell degeneration. Scale bar: 10 µm.

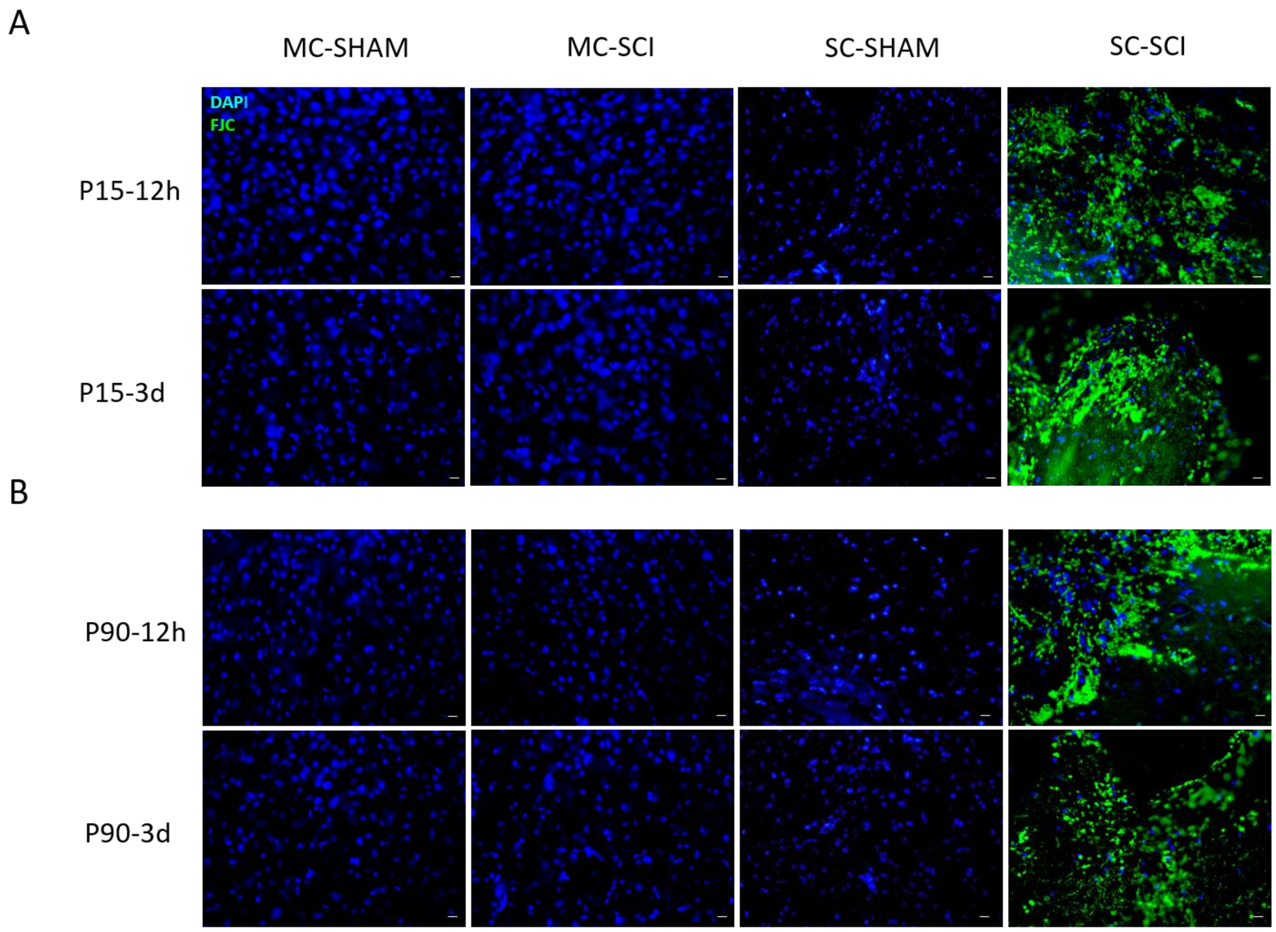

Figure S3. Relative expression of miR-7b-3p transcript level upon mimic overexpression in N2a cells.

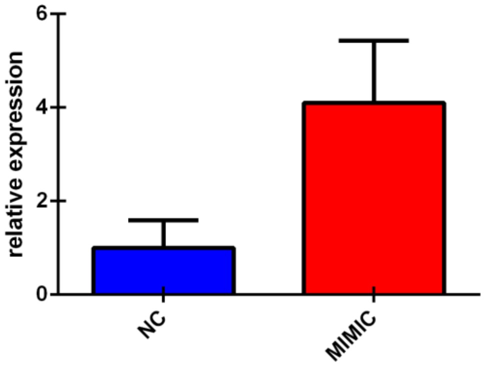

Table S1: the main functions of the seven validated target genes of miR-7a-2-3p and possible targets of miR-7b-3p

	Experimental group(s)	Functions	References
**Zdhhc9**	P15-3d	• Mutations associated with epilepsy, intellectual disability, SMA and ALS<br>• It regulates localization of axon survival factors ;required for axon growth and maintenance after injury<br>• Control of synaptic function assembly and neurotransmitter receptors	• Bathelt J. Et al., 2016; Zhang Z. et al., 2008; Pol Andres-Benito et al., 2017<br>• Milde S and Coleman MP, 2014; Gilley J and Coleman MP 2010; Xia T. et al., 2014; Holland SM and Thomas GM, 2017; Pinner AL. et al., 2016<br>• Han J. Et al., 2015; Dalva MB 2009; Naumenko VS and Ponimaskin E 2018; Thomas GM, 2012
**Wipf2**	P15-3d<br>P90-3d	• Essential for neurite outgrowth/extension, branching, differentiation and cytoskeletal rearrangement<br>• Increased in epilepsy and Alzheimer's disease	• Kakimoto T. et al., 2004, Miki H. et al., 1996, Takenawa T. et al., 2005, Banzai Y. Et al., 2000, Kitamura Y. Et al., 2003<br>• Xiao F. et al., 2008, Kitamura Y. Et al., 2003
**Prkcb**	P90-3d	• Still not clear the implicatation in neuronal maturation, proliferation and differentiation; downregulated in ischemia<br>• Involved in apoptosis and cell death	• Kaur P et al., 2014; Guo S. et al., 2012<br>• Genini S et al., 2010
**Pfn2**	P90-3d	• Preferentially distributed to shaft of growing axons; regulation of actin stability during neuritogenesis<br>• Its lack reduces dendritic complexity, spine numbers and block of synaptic actin depolimerization<br>• Decreased levels in CMT2 (Charcot-Marie-Tooth disease) and increased in SMA	• Wang YY. Et al., 2014, Da Silva JS et al., 2003<br>• Michaelsen K et al., 2010, Pilo Boyl P. et al., 2007<br>• Juneja M et al., 2018, Bowerman M. et al., 2009

Table S2: the main functions of the predicted target genes of miR-7b-3p that were confirmed to be downregulated at the cortical level of SCI mice by RT-PCR

	Experimental group(s)	Functions	References
**APC**	P90-12h	• Induction of neurite extension and lamellipodia formation<br>• Promotion of neurite and axon outgrowth of hippocampal neurons<br>• Regulation of microtubule organization at the basal cortex<br>• Its lack causes cerebral cortex disorganization, cerebellum deficit, impaired neurogenesis and locomotor impairment	• Elbaz B et al., 2016<br>• Votin V et al., 2005<br>• Reilein A. et al., 2005<br>• Shintani T. et al., 2012, Koshimizu H. et al., 2011, Imura T. et al. 2010
**Unc5c**	P90-12h	• Control of axon trajectories<br>• Its mutations cause abnormal axon invasion in spinal cord, hindbrain and DRG	• Masuda T. et al., 2008, Furne C. et al., 2008<br>• Dillon AK. Et al., 2007, Watanabe K. Et al., 2006, Laumonnerie C. et al., 2014, Kim D. and Ackerman SL., 2011
**G6PC**	P90-12h<br>P15-3d	• Expressed in murine cortex and hipotalamus	• Goh BH. Et al., 2006
**Ntrk2**	P90-12h<br>P15-3d	• Development and maturation of CNS and manteinance of aminergic neuronal population<br>• It increases the gain rates of spines and sinaptic boutons<br>• Promotion of neurogenesis<br>• Promotion of reactive axonal sprouting	• Almoguera B. et al., 2019, Prabhakaran N. et al., 2018, Sahu MP. Et al., 2019<br>• Perz-Rando M. et al., 2018<br>• Wei Z. et al., 2015<br>• Aungst S. et al., 2013
**Ptrh2**	P90-12h	• Manteinance of cell survival in developing brain	• Hu H. et al., 2014
**Tle**	P90-12h	• Negative regulator of postmitotic neuronal differentiation<br>• Promotion of interneuron generation in spinal cord	• Yao J. Et al., 2000, Buscarlet M et al., 2009 and 2008, Nuthall HN. Et al., 2004<br>• Todd KJ et al., 2012

	Experimental group(s)	Functions	References
**Ablim1**	P15-3d	• Actin binding domain that acts on axon guidance and remodelling<br>• Intracellular guidance cue<br>• Mutations of its homolgue (unc-115) causes defects in axon guidance	• Erkmann L.et al., 2000, Lu C. et al., 2003<br>• McIntyre JC. Et al., 2010<br>• Lundquist EA. et al., 1998
**Arhgef12**	P15-3d	• It is part of a regulatory network involved in axon regeneration<br>• Upregulated after SCI, associated with tissue protection and neural development	• Su LN. Et al., 2018<br>• Chang YW. Et al., 2009
**Dpysl5/CRMP5**	P15-3d	• It regulates dendritic development and differentiation<br>• Activated by Sox5 (a modulator of neurite outgrowth)<br>• It regulates axon-Schwann cell cooperation<br>• Inhibition of dendrite and neurite outgrowth	• Bretin S. et al., 2005; Qin L. et al., 2017; Naudet N. et al., 2018; Yamashita N. et al., 2011<br>• Naudet N. et al., 2018; McLaughlin D. et al., 2008<br>• Camdessanché JP. Et al., 2012<br>• Brot S. et al., 2014; Brot S. et al., 2010
**PAK7**	P15-3d	• Required for growth cone steering, neurogenesis and dorsal closure (in Drosophila)<br>• Induction of neurite outgrowth , filopodia formation and synaptic vescicle traffiking	• Harden N. et al., 1996; Hing H. et al., 1999; Melzig J. Et al., 1998<br>• Strochlic Tl. Et al., 2012; Derek WM. Et al., 2014; Dan C. et al., 2002; Matenia D. et al., 2005; Timm T. et al., 2006
**PAK6**	P15-3d	• Promotion of neurite outgrowth<br>• It increases after traumatic brain injury	• Civiero L. et al., 2015 and 2017<br>• Chen XD. Et al., 2011
**Arpc2**	P15-3d	• Promotion of cell migration and motility of OPCs (oligodendrocyte precursors)	• Li Y. Et al., 2015

	Experimental group(s)	Functions	References
**Unc5d**	P15-3d	• Control of radial migration of pyramidaland cerebellar neurons<br>• Promotion of axon outgrowth in C. Elegans spinal cord<br>• Induction of neurite outgrowth, axon branching and extension	• Seiradake E. et al., 2014; Yamagishi S. et al., 2011; Miyoshi G. and Fishell G., 2012; Alcantara S. et al., 2000
**Ephb1**	P15-3d	• Inhibition of allodynia and hyperalgesia after nerve injury<br>• Promotion of synaptic plasticity in spinal cord and brain<br>• Its repulsive action controls axon trajection in retina, spinal cord and brain<br>• Its lack causes impairments in neurogenesis<br>• Upregulated after brain injury	• Mey Yang et al., 2018; Xia WS. Et al., 2014; Song XY. Et al., 2008<br>• Liu S. et al., 2015; Liu WT. Et al., 2009; Slack S. et al., 2008; Nolt MJ. Et al., 2011; Kayser MS. et al., 2006<br>• Luria V. et al., 2008; Lee R. et al., 2008; Carmichael ST. et al., 2005; Imondi R. et al., 2000; Robichaux MA. Et al., 2016 and 2014; Bouché E. et al., 2013; Williams SE. Et al., 2006; Lambot MA. Et al., 2005; Pak W. Et al., 2004; Conover JC. Et al., 2000<br>• Bestman JE. Et al., 2015; Chumley MJ. Et al., 2007<br>• Biervert C. et al., 2001
**Itgb3**	P15-3d	• It is a cofactor of SERT (serotonin transporter) whose level decreases after SCI<br>• Decreased in depression disorders	• Husch A. et al., 2012<br>• Rzezniczek S. et al., 2016; Fabbri C. et al., 2015; Probst-Schendzielorz K. Et al., 2015
**Nos3**	P15-3d	• Protective effect after stroke<br>• Its inhibition confers post-ischemic protection and coincides with active neurogenesis<br>• Induction of neuronal death and it is required to inhibit neural cell proliferation<br>• It is increased in SCI, enhancing astrocytes and microglia viability	• Mallei A. et al., 2018<br>• Bastian C. et al., 2018; Chavez GG. Et al., 2017<br>• De la Monte SM. Et al., 2003 and Jezierski A. et al., 2012<br>• Jiang ZS. Et al., 2018; Ma S. et al., 2017; Su YF. Et al., 2015 and Liu C. et al., 2002
